# Camptocormia and Other Orthopedic Compromise Dominating Mitochondrial Disorder: A Case Report

**DOI:** 10.7759/cureus.11888

**Published:** 2020-12-03

**Authors:** Josef Finsterer, Subhankar Chatterjee, Ritwik Ghosh

**Affiliations:** 1 Neurology, Krankenanstalt Rudolfstiftung, Vienna, AUT; 2 Department of General Medicine, Rajendra Institute of Medical Sciences, Jharkhand, IND; 3 Department of General Medicine, Burdwan Medical College and Hospital, Burdwan, IND

**Keywords:** mitochondrial disorder, multisystem, mtdna, phenotype, orthopedic

## Abstract

Objectives: Camptocormia and other orthopedic abnormalities have been only rarely reported as a phenotypic manifestation of a mitochondrial disorder (MID). Here we present an MID patient with multiple orthopedic abnormalities dominating the phenotype.

Case report: The patient is a 55-year-old male in whom MID was diagnosed at age 34 upon clinical presentation, muscle biopsy, and biochemical investigations. Phenotypically, he manifested with multisystem disease including the brain (mental retardation, epilepsy, sleep disorder, cerebellar atrophy), eyes (cataract, myopia), ears (hypoacusis), heart (hypertrophic, cardiomyopathy, QT-prolongation, left anterior hemiblock, noncompaction), intestines (hepatopathy, cholecystolithiasis), muscle (myopathy), peripheral nerves (neuropathy), and the bone marrow (anemia). Additionally, there was facial dysmorphism (upslanting palpebral fissures, hypertelorism, protruding bulbs) and multiple orthopedic abnormalities, including camptocormia in the absence of axial myopathy, barrel thorax, gibbus, genu valga, knee contractures, bilateral gonarthrosis, bilateral ankle arthroses, and outwardly rotated feet. These abnormalities were complicated by wedge vortex, vertebral stenosis, and coxarthrosis requiring right hip endoprosthesis. His mother manifested with a largely different phenotype.

Conclusions: An MID can manifest phenotypically with orthopedic abnormalities, which may dominate the phenotype. According to this case, orthopedic abnormalities in a MID can be unrelated to the severity of myopathy and intrafamilial phenotypic variability can be high in a MID.

## Introduction

It is well established that mitochondrial disorders (MIDs) are frequently multisystem disorders [1] and that the phenotype of a maternally transmitted MID may vary considerably within a family and between families [2]. Orthopedic abnormalities, in particular camptocormia (bent spine syndrome), have been only rarely reported as a phenotypic manifestation of a MID [3]. Here we report an MID patient with predominant orthopedic involvement and high intrafamilial phenotypic variability.

## Case presentation

**Figure 1:**
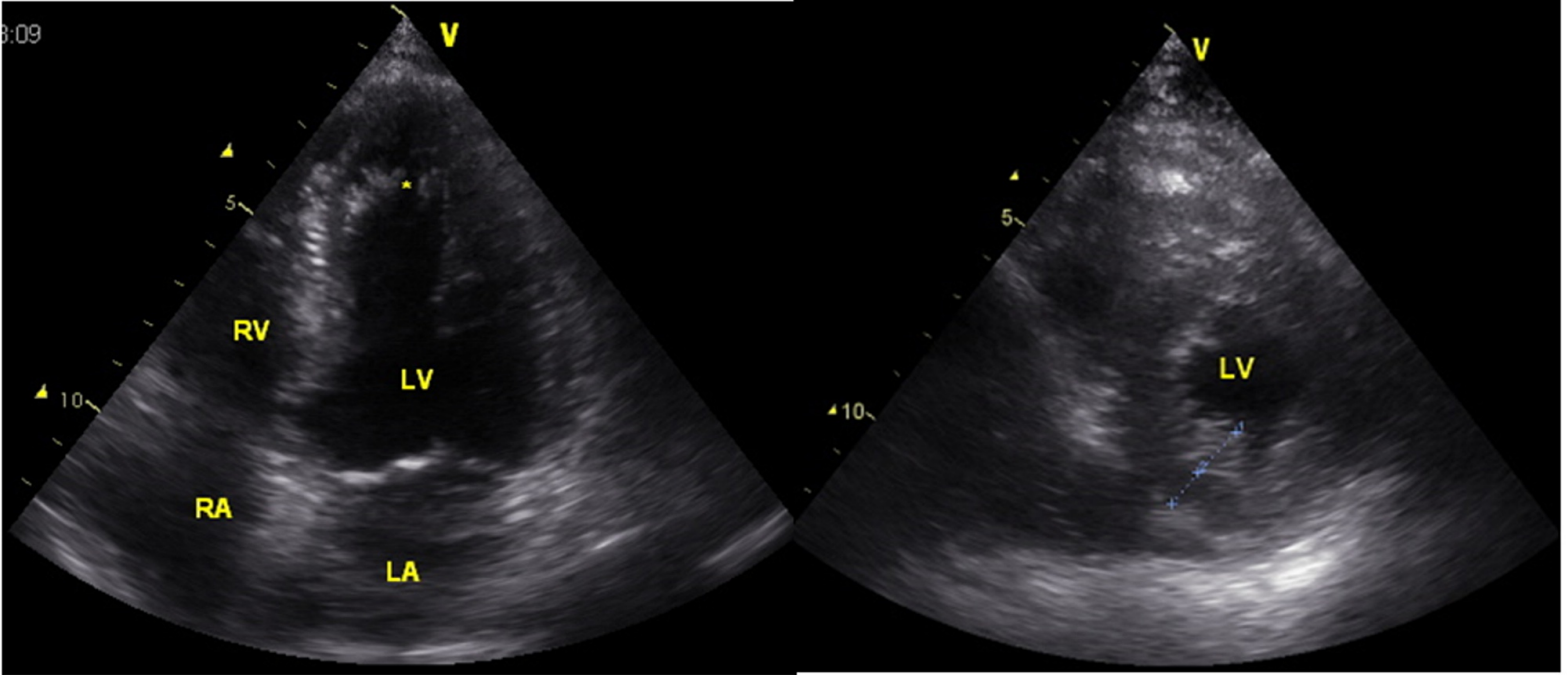
Transthoracic echocardiography 4-chamber view showing left ventricular hypertrabeculation (LVHT)/left ventricular noncompaction cardiomyopathy (LVNC) of the left ventricular apex (*) and the adjacent septal and lateral myocardium (left panel). In the parasternal axial view a two-layered structure of the myocardium (inner layer is echodens and hypertrabeculated and the outer layer is homogenous) distal to the mitral leaflet is visible (right panel).

**Figure 2:**
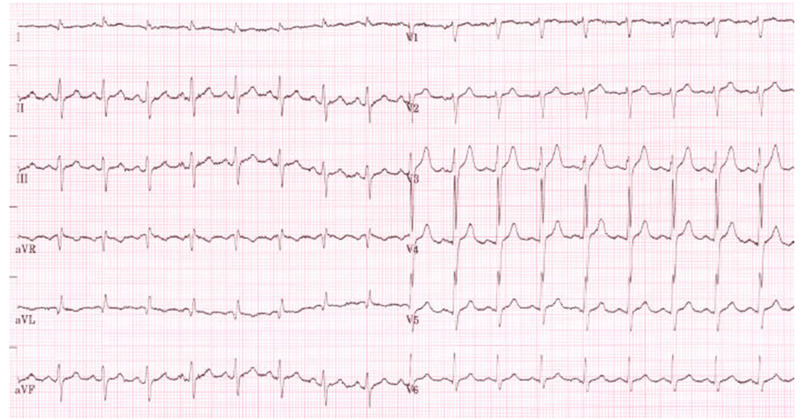
Electrocardiogram (ECG) recording at age 49 showing a prolonged QTc-interval of 459ms

The index patient is a 55-year-old Caucasian male, height 160cm, weight 72kg, with a multisystem MID diagnosed upon clinical presentation, immune-histo-chemical, and biochemical investigations of a muscle biopsy at age 34. His history was noteworthy for mental retardation, epilepsy since age 12, cataract surgery, hypoacusis, vitamin D deficiency, hypertrophic cardiomyopathy (hCMP), left ventricular hypertrabeculation (LVHT)/noncompaction (Figure 1), QT-prolongation (Figure 2), left anterior hemiblock (LAH), anemia, cholecystolithiasis, sleep disorder, hepatopathy, hyperlipidemia, benzodiazepine misuse, myopathy, prostate hyperplasia, right total hip endoprosthesis, and iron deficiency (Table 1). Since at least age 47 camptocormia became apparent. At age 48 he experienced a deep venous thrombosis after left tibial fracture. At age 51 he had undergone surgery for right-sided scrotal hernia. Seizures were well controlled with a seizure frequency of about two generalised tonic-clonic seizures per year and several focal seizures.

**Table 1:** Phenotypic similarities and discrepancies between mother and son Uk: unknown, hCMP: hypertrophic cardiomyopathy, HRT: hip replacement therapy

		Mother	Son
CNS			
	Mental retardation	no	yes
	Epilepsy	no	yes
	Leukoencephalopathy	yes	no
	Sleep disorder	uk	yes
	Atrophy	yes (global)	yes (cerebellar)
Eyes			
	Cataract	yes	yes
Ears			
	Hypoacusis	no	yes
Endocrine			
	Prostate hyperplasia	no	yes
	Diabetes	yes	no
	Hyperlipidemia	yes	yes
	Short stature	yes	yes
Heart			
	hCMP	no	yes
	QT-prolongation	no	yes
	Noncompaction	no	yes
	Left anterior hemiblock	no	yes
	Arterial hypertension	yes	no
Gastrointestinal			
	Hepatopathy	no	yes
	Cholecystolithiasis	no	yes
Bone marrow			
	Anemia	no	yes
Muscle			
	Myopathy	yes	yes
Peripheral nerves			
	Neuropathy	yes (diabetic)	yes
Orthopedic			
	Camptocormia	no	yes
	Genu valga	no	yes
	Contractures of knees	no	yes
	Barrel thorax	no	yes
	Outwardly rotate feet	no	yes
	Wedge vortex	no	yes
	Lumbar vertebral stenosis	no	yes
	Coxarthrosis (HRT)	no	yes
	Listhesis L3/4	yes	no
Others			
	Iron deficiency/overload	yes (overload)	yes (deficiency)
	Right ingual/scrotal hernia	no	yes
	Gibbus	no	yes
	Left tibial fracture	no	yes
	Deep venous thrombosis	yes	yes
	Alopecia	yes	no
	Madarosis	yes	no
	Vitamin-D deficiency	no	yes
	Polyarthralgias	yes	no
	Hyperuricemia	yes	no
		Mother	Son
CNS			
	Mental retardation	no	yes
	Epilepsy	no	yes
	Leukoencephalopathy	yes	no
	Sleep disorder	uk	yes
	Atrophy	yes (global)	yes (cerebellar)
Eyes			
	Cataract	yes	yes
Ears			
	Hypoacusis	no	yes
Endocrine			
	Prostate hyperplasia	no	yes
	Diabetes	yes	no
	Hyperlipidemia	yes	yes
	Short stature	yes	yes
Heart			
	hCMP	no	yes
	QT-prolongation	no	yes
	Noncompaction	no	yes
	Left anterior hemiblock	no	yes
	Arterial hypertension	yes	no
Gastrointestinal			
	Hepatopathy	no	yes
	Cholecystolithiasis	no	yes
Bone marrow			
	Anemia	no	yes
Muscle			
	Myopathy	yes	yes
Peripheral nerves			
	Neuropathy	yes (diabetic)	yes
Orthopedic			
	Camptocormia	no	yes
	Genu valga	no	yes
	Contractures of knees	no	yes
	Barrel thorax	no	yes
	Outwardly rotate feet	no	yes
	Wedge vortex	no	yes
	Lumbar vertebral stenosis	no	yes
	Coxarthrosis (HRT)	no	yes
	Listhesis L3/4	yes	no
Others			
	Iron deficiency/overload	yes (overload)	yes (deficiency)
	Right ingual/scrotal hernia	no	yes
	Gibbus	no	yes
	Left tibial fracture	no	yes
	Deep venous thrombosis	yes	yes
	Alopecia	yes	no
	Madarosis	yes	no
	Vitamin-D deficiency	no	yes
	Polyarthralgias	yes	no
	Hyperuricemia	yes	no
CNS			
	Mental retardation	no	yes
	Epilepsy	no	yes
	Leukoencephalopathy	yes	no
	Sleep disorder	uk	yes
	Atrophy	yes (global)	yes (cerebellar)
Eyes			
	Cataract	yes	yes
Ears			
	Hypoacusis	no	yes
Endocrine			
	Prostate hyperplasia	no	yes
	Diabetes	yes	no
	Hyperlipidemia	yes	yes
	Short stature	yes	yes
Heart			
	hCMP	no	yes
	QT-prolongation	no	yes
	Noncompaction	no	yes
	Left anterior hemiblock	no	yes
	Arterial hypertension	yes	no
Gastrointestinal			
	Hepatopathy	no	yes
	Cholecystolithiasis	no	yes
Bone marrow			
	Anemia	no	yes
Muscle			
	Myopathy	yes	yes
Peripheral nerves			
	Neuropathy	yes (diabetic)	yes
Orthopedic			
	Camptocormia	no	yes
	Genu valga	no	yes
	Contractures of knees	no	yes
	Barrel thorax	no	yes
	Outwardly rotate feet	no	yes
	Wedge vortex	no	yes
	Lumbar vertebral stenosis	no	yes
	Coxarthrosis (HRT)	no	yes
	Listhesis L3/4	yes	no
Others			
	Iron deficiency/overload	yes (overload)	yes (deficiency)
	Right ingual/scrotal hernia	no	yes
	Gibbus	no	yes
	Left tibial fracture	no	yes
	Deep venous thrombosis	yes	yes
	Alopecia	yes	no
	Madarosis	yes	no
	Vitamin-D deficiency	no	yes
	Polyarthralgias	yes	no
	Hyperuricemia	yes	no

Clinical neurologic exam revealed short stature, hypertelorism, myopia, bilateral ptosis (left > right), signe des cils bilaterally, bulb protrusion bilaterally, upslanting palpebral fissures, positional nystagmus when looking in any direction, cognitive impairment, hypoacusis, a barrel thorax, gibbus, camptocormia, generally reduced tendon reflexes on the upper and lower limbs, diffuse wasting, dysdiadochokinesia, mild postural tremor, mild weakness for foot extension bilaterally (M5-), ataxia of the lower limbs, genu valga, outwardly rotated feet, and flexion contractures of the knees with right-sided predominance.

Creatine-kinase (CK) was repeatedly elevated. Serum lactate was elevated to 2.5 mmol/l (n, <2.2 mmol/l). The lactate stress test was abnormal. Nerve conduction studies were indicative of axonal polyneuropathy. MRI of the lumbar spine revealed a wedge vortex L3, vertebral stenosis L2/3, and lumbar osteochondrosis. Muscle biopsy from the right gastrocnemius muscle revealed subsarcolemmal accumulation of mitochondria, ragged red fibers, tubular-shaped mitochondria, and bizarre cristae proliferation. Biochemical investigations of the muscle homogenate revealed a combined complex-I and complex-IV defect. His last medication included lamotrigine 400 mg/d, levetiracetam 1000 mg/d, lacosamide 250 mg/d, and vitamin D.

The 78-year-old mother of the index patient had a history of arterial hypertension, diabetes, hyperlipidemia, polyarthralgias due to polyarthrosis, deep venous thrombosis, recurrent leg edema, phlebitis, hyperuricemia, and listhesis L3/4. She had surgery of the right shoulder after a trauma, cataract surgery bilaterally, surgery for carpal tunnel syndrome bilaterally, and veins tripping four times. She refrained from being investigated neurologically during years despite frequently accompanying her son to his regular neurological visits. At the age of 74 she sought neurological advice for the first time after nerve conduction studies, carried out for sensory disturbances of the feet, had revealed sensori-motor polyneuropathy.

Clinical exam of the mother revealed short stature (164 cm), alopecia, madarosis, dysarthria, diffuse weakness of the left upper limb, hypoesthesia of the left upper limb, reduced triceps tendon reflexes bilaterally, dysdiadochokinesia, dysmetria bilaterally, reduced tendon reflexes on the lower limbs, and discrete wasting of the thighs. The hemoglobin A1c (HbA1c) value was 7.3 % (n, 4-6%). Triglycerides were 313 mg/dL (n, <150 mg/dL). Serum iron was elevated to 187mg/dl (n, 27-145 mg/dl), the CK was 224 U/L (n, <170 U/L). There was mild lactic acidosis. Cerebral MRI showed gliotic spots bilaterally including the basal ganglia and diffuse atrophy. Nerve conduction studies revealed a sensori-motor polyneuropathy. Her last medication included simvastatin, enalapril, acetyl-salicylic acid, vitamins, and antidiabetics. She refused to undergo muscle biopsy. Phenotypic features the index patient and his mother had in common included cerebral atrophy, myopathy, neuropathy, cataract, hyperlipidemia, short stature, disturbed iron metabolism, and deep venous thrombosis (Table 1). Other family members had anginal chest pain (brother, father, mother), subarachnoidal bleeding (father), and cholecystolithiasis (grandmother from the mother's side).

## Discussion

The presented patient is unique for the described combination of orthopedic abnormalities and for predominant orthopedic involvement in a multisystem MID affecting the brain (cognitive impairment, epilepsy, ataxia, cerebellar atrophy, sleep disorder), eyes (cataract, myopia), ears (hypoacusis), endocrine organs (short stature, hyperlipidemia), heart (QT-prolongation, LAH, LVHT, hCMP), peripheral nerves (polyneuropathy), muscle (myopathy, ptosis, camptocormia), and bone marrow (anemia). Additionally, the patient manifested with facial dysmorphism (hypertelorism, upslanting palpebral fissures, protruding bulbs) and orthopedic manifestations, including camptocormia, genu valga, barrel thorax, gibbus, and inwardly rotated feet. His mother manifested only in the endocrine organs, eyes, and muscle. Both had only a few phenotypic features in common.

The index patient is noteworthy for the orthopedic abnormalities described above which have not been reported in combination. Orthopedic abnormalities previously reported in MID include scoliosis [4], genu valga [5], gibbus [6], pectus carinatum (pigeon breast) [7], brachydactylia [8], foot deformities [9], skeletal dysplasia [10], Kniest dysplasia [11], and camptocormia (Table 2) [3]. Inwardly rotated feet and barrel thorax have not been reported in MIDs. Camptocormia has been only rarely reported in MIDs (Table 2), most frequently in association with axial myopathy [12]. Affection of the axial muscles in the index case was not clinically evident but may have been present subclinically. Most likely camptocormia in the index patient was thus also due to subclinical weakness of the axial muscles.

**Table 2:** Camptocormia in mitochondrial disorders f: female, m: male, ns: not specified

Patient/Age	Sex	Mutated gene	Reference
55	m	ns	[index case]
71	f	MT-TV	[3]
53	m	POLG1	[13]
58	f	POLG1	[13]
71	f	RRM2B	[13]
79	m	POLG1	[14]
73	f	tRNA(Phe)	[15]
78	f	ns	[16]
Ns	ns	ns	[17]
Ns	ns	ns	[12]

Whether orthopaedic abnormalities in the index case were primary or secondary, remains speculative. Since there was no clinical evidence for weakness of the axial musculature and only mild weakness for foot extension, being attributed to polyneuropathy, we interpreted most of them as primary. Primary orthopedic disease may pathogenetically derive from abnormal embryonic development of the bones or cartilage. An explanation for the orthopaedic abnormalities in MIDs could be involvement of the hypoxic tissue cartilage in the metabolic breakdown [18]. Since skeletal growth is driven by expansion of cartilage in the growth plate [18], it is conceivable that disturbed cartilage proliferation may lead to orthopedic compromise during intrauterine or childhood development. Whether some of the orthopedic abnormalities (genu valga, gibbus) were attributable to vitamin D deficiency remains speculative, since it was well substituted during the last years. Whether the barrel thorax was due to cardiac involvement remains speculative but is rather unlikely given the high number of MID patients with cardiac involvement but without this thorax deformity described in the literature. Facial dysmorphism is a common finding in MIDs, which has been previously reported [11,19]. Most of the other phenotypic features are common in MIDs, except for camptocormia, which has been reported only in POLG1 mutation carriers.

Though not genetically confirmed, maternal transmission of the MID in the presented family is quite likely. Arguments for maternal transmission are that the mother had developed features of a MID, that mitochondrial DNA (mtDNA)-related MIDs are transmitted via the maternal line in 75% of the cases. and that none of the males in this family had transmitted the disease. The variable phenotype between mother and son does not exclude maternal transmission, as intrafamilial phenotypic variability in MID families can be high [2,20].

In conclusion this case shows that a MID can manifest phenotypically with orthopedic abnormalities, which may dominate the phenotype of a maternally transmitted MID. According to this case, orthopedic disease in a MID can be unrelated to the severity of myopathy and intrafamilial phenotypic variability can be high in a MID.

## Conclusions

This case shows that a MID can manifest phenotypically with orthopedic abnormalities, which may dominate the phenotype of a maternally transmitted MID. According to this, orthopedic disease in a MID can be unrelated to the severity of myopathy and intrafamilial phenotypic variability can be high in a MID.
